# The Effect of Red Meat Consumption on Circulating, Urinary, and Fecal Trimethylamine-N-Oxide: A Systematic Review and Narrative Synthesis of Randomized Controlled Trials

**DOI:** 10.1016/j.advnut.2025.100453

**Published:** 2025-05-24

**Authors:** Fatemeh Jafari, Janhavi J Damani, Kristina S Petersen

**Affiliations:** Department of Nutritional Sciences, Pennsylvania State University, University Park, PA, United States

**Keywords:** red meat, pork, beef, trimethylamine N-oxide, systematic review, clinical trials, humans

## Abstract

Cardiovascular concerns exist about the effect of red meat on circulating concentrations of trimethylamine N-oxide (TMAO), an emerging cardiovascular disease risk factor. The aim was to conduct a systematic review of randomized controlled trials (RCTs) to evaluate the effect of higher red meat intake, compared with lower intake, on circulating, urinary, and fecal TMAO concentrations in generally healthy adults and/or adults with stable chronic diseases. A systematic literature search was conducted using PubMed, the Cochrane Collaboration Library, and Web of Science. RCTs examining the effect of a ≥7-d dietary intervention featuring red meat on urinary, fecal, and/or circulating (plasma or serum) concentrations of TMAO in adults (≥18 y) were included. Eligible trials had a comparator group/condition that was exposed to a dietary intervention for ≥ 7 d lower in red meat and featuring white meat, fish, eggs, dairy, or plant-based protein sources. In total, 375 publications were identified. Fifteen publications reporting the results of 13 RCTs (*n* = 553; median duration 28 d), including 15 diet comparisons, were eligible. In 6 comparisons, higher circulating or urinary TMAO concentrations were observed after higher red meat intake (∼71–420 g/d) compared with comparator conditions lower in red meat. In 7 comparisons, no differences in serum/plasma TMAO concentrations were observed with higher red meat-containing diets (∼60–156 g/d) compared with diets lower in red meat. Two comparisons showed that consuming higher red meat diets lowered TMAO concentrations after 28 d compared with lower red meat diets containing seafood. In short-term studies (median duration of 28 d), higher red meat intake had inconsistent effects on circulating and urinary TMAO concentrations. Further high-quality research on red meat-related TMAO modulation, including effect magnitude and clinical relevance, is needed. This study was registered at Prospective Register of Systematic Reviews (PROSPERO) as CRD42023396799.

**Statements of Significance**

This systematic review summarizes evidence on the effect of higher red meat intake, compared with lower intake of red meat, on circulating, urinary, and fecal concentrations of trimethylamine N-oxide (TMAO) in generally healthy adults and/or adults with stable chronic diseases. Higher red meat intake had inconsistent effects on TMAO concentrations, which may be partly related to differences in clinical trial methodology, interindividual variability in diet-related TMAO modulation, and/or the overall healthfulness of the red meat-containing diet.

## Introduction

Cardiovascular disease (CVD) is the leading cause of death globally and in the United States [1]. Observational studies show that a higher intake of red meat, especially processed meat is associated with a greater risk of CVD and cardiovascular mortality [[2], [3], [4], [5]]. However, causality cannot be inferred from epidemiological data because of potential confounding from lack of consideration for red meat type and fat content, background diet quality, and other lifestyle behaviors that accompany red meat consumption. Nonetheless, the documented relationship between red meat intake and CVD may, in part, be explained by trimethylamine N-oxide (TMAO), an emerging risk factor for CVD linked to red meat consumption [6,7].

Observational studies show plasma TMAO concentration is an independent risk factor of CVD after adjustment for traditional CVD risk factors [[8], [9], [10]]. In addition, epidemiologic evidence suggests that TMAO partially mediates the positive association between red meat intake and atherosclerotic cardiovascular disease (ASCVD) risk [11]. An analysis from the Cardiovascular Health Study, including 3931 adults older than 65 y, showed that higher consumption of unprocessed red meat [hazard ratio (HR) 1.15; 95% confidence interval (CI): 1.01, 1.30], processed meat (HR: 1.11; 95% CI: 0.98, 1.25), and total meat (HR 1.22; 95% CI: 1.07, 1.39) was associated with higher ASCVD risk, which was partially (8%–11%) mediated by TMAO, and its intermediates butyrobetaine and crotonobetaine [11].

TMAO is endogenously produced from diet-derived L-carnitine, choline, and phosphatidylcholine by gut microbiota-dependent transformation to trimethylamine (TMA) followed by transformation to TMAO by hepatic flavin-containing monooxygenase 3. Oral supplementation with L-carnitine significantly increases circulating TMAO concentration [12]. Although high doses of supplemental L-carnitine have been shown to increase TMAO concentrations acutely and after chronic ingestion for ≤2 mo [8,13], these observations cannot be extrapolated to dietary sources of L-carnitine such as red meat because of potential differences in absorption. Supplemental L-carnitine has a much lower absorption rate (5%–18%) compared with dietary L-carnitine, which is absorbed much more efficiently (54%–87%) [14]. Therefore, supplemental L-carnitine results in larger amounts of unabsorbed L-carnitine available for gut microbiota metabolism, which may lead to differences in TMAO production with dietary L-carnitine intake compared with supplements [15]. Clinical trials investigating the effects of red meat intake on TMAO concentrations are limited and have shown mixed results, and to date no evidence of synthesis is available. Therefore, the aim was to conduct a systematic review of randomized controlled trials (RCTs) to evaluate the effect of higher red meat intake, compared with lower intake, on circulating, urinary, and fecal TMAO concentrations in generally healthy adults and/or adults with stable chronic diseases.

## Methods

A systematic review of RCTs was performed to examine the effect of higher red meat intake, compared with lower intake of red meat and/or intake of other animal-derived protein sources and/or nonanimal derived protein sources, on circulating, urinary, and fecal concentrations of TMAO in generally healthy adults. The systematic review was conducted according to the Cochrane Collaboration Handbook [16]. The protocol was prospectively registered at the PROSPERO (identifier: CRD42023396799).

### Search strategy

A systematic literature search was conducted using PubMed, the Cochrane Collaboration Library, and Web of Science from the index date of each database through 15 February, 2023, and updated on 1 August, 2024 (Supplemental Table 1). All authors (FJ, JJD, KSP) were involved in screening the titles/abstracts of articles identified in the search using Rayyan (Qatar Computing Research Institute). Each search result was reviewed independently by ≥2 reviewers. All full texts were reviewed in duplicate (FJ, JJD, KSP). Disagreements were resolved by consensus.

### Selection criteria

RCTs that examined the effect of a ≥7-d dietary intervention featuring red meat (i.e., beef, pork, lamb) on circulating (plasma or serum), urinary, and/or fecal TMAO concentration were included. Eligible trials included generally healthy adults (≥18 y) and/or adults with stable chronic diseases. In addition, eligible trials had a comparator group/condition that was exposed to a dietary intervention for ≥7 d that was lower in red meat and featured commonly consumed protein sources including white meat (i.e., chicken, turkey, other poultry), fish, eggs, dairy or plant-based proteins. These comparators were selected to enable assessment of the relative effects of higher red meat intake on TMAO compared with protein sources commonly consumed instead of red meat. Although these comparators contain varying amounts of TMAO precursors and preformed TMAO, these protein sources are typically consumed as a replacement for red meat and therefore have the greatest public health relevance. Nonrandomized studies, quasi-experimental studies, observational studies, narrative reviews, systematic reviews, meta-analyses, preclinical studies, case series, case reports, protocol papers, and conference proceedings were excluded. Studies that had a dietary supplement as the comparator or a concomitant intervention that did not enable the effects of red meat to be estimated were excluded. Studies including pregnant and lactating females, those with serious unstable medical conditions (e.g., cancer, chronic kidney diseases), and medical conditions known to affect TMAO metabolism (e.g., impaired kidney function) were excluded. Only studies published in English were eligible for inclusion.

### Data extraction

Data were extracted from the eligible studies by 1 author (FJ) and entered into a standardized spreadsheet. A second author checked the extracted data (JJD, KSP). The following items were extracted: study design (parallel/crossover); study characteristics (health status, sample size, percentage of male participants, mean age and mean BMI of participants), intervention and comparator characteristics (duration, type of protein, dosage, meat species, processing, leanness, implementation method), and background diet characteristics (type, macronutrient and micronutrient profile, intake of food groups, TMAO precursor content). TMAO concentration (reported as mean or median and variance) prior to each dietary intervention and after each dietary intervention was extracted. The mean difference (and variance) in TMAO between the dietary interventions was extracted when reported. In addition, the type of biological sample used for TMAO measurement, and the laboratory assay method were also extracted. Where reported, choline and betaine concentrations were also extracted. A priori, it was decided that a meta-analysis would not be conducted because of the expected methodological heterogeneity across the eligible studies. Therefore, a systematic review with a narrative synthesis of the results from the primary analysis as reported by the authors was conducted.

### Risk of bias

Using the Cochrane Risk of Bias 2 Tool [16], risk of bias in the included studies was assessed in duplicate (FJ, JJD, KSP). Authors assessed whether there were some concerns, low or high risk of bias arising from the randomization process, period and carryover effects (crossover studies only), deviations from the intended interventions, missing outcome data, measurement of the outcome, and selection of the reported result. Disagreements were resolved by consensus. Grading of Recommendations Assessment, Development, and Evaluation (GRADE) was used to evaluate evidence certainty, considering risk of bias, inconsistency, indirectness, imprecision, and publication bias [17].

## Results

### Search summary

**FIGURE 1:**
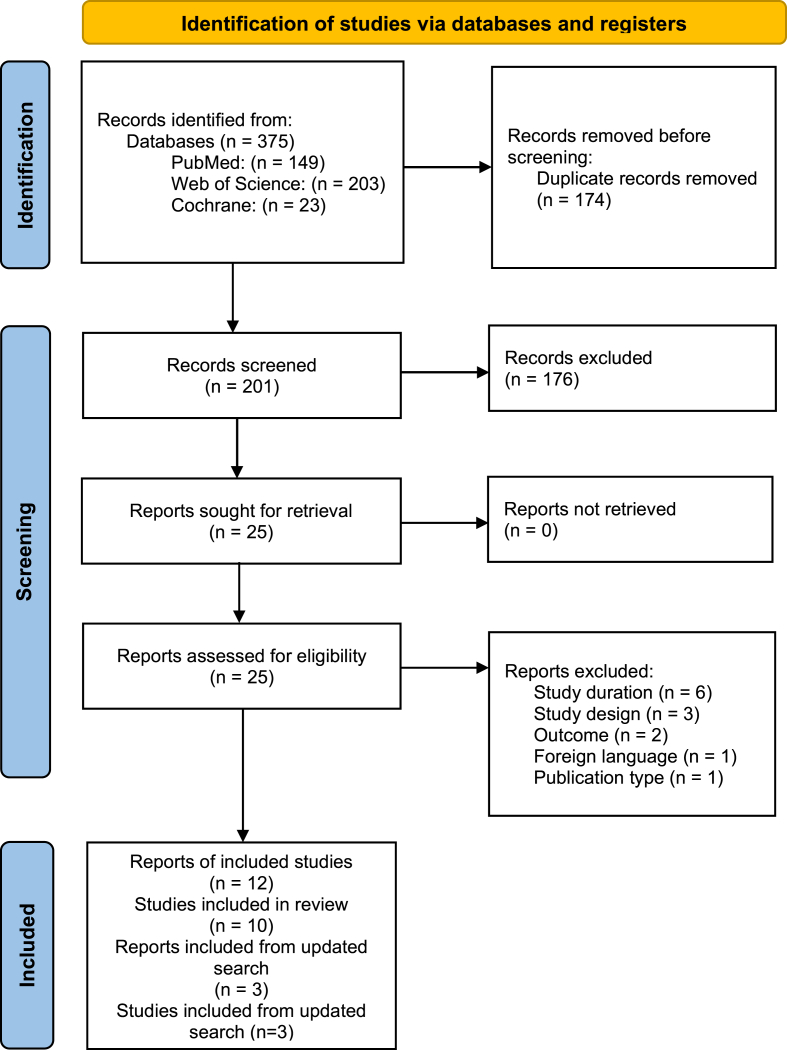
PRISMA flow diagram for included publications.

The search strategy identified 375 publications. After removing the duplicates, 173 articles were eligible for screening. After title and abstract screening, 28 articles were eligible for full text screening and 12 articles met all the inclusion criteria (Figure 1). Three additional publications were identified when the search was updated on 1 August, 2024.

### Trial characteristics

Results from 13 unique RCTs reported in 15 eligible publications [[18], [19], [20], [21], [22], [23], [24], [25], [26], [27], [28], [29], [30], [31], [32]] were included (Table 1) [[18], [19], [20], [21], [22], [23], [24], [25], [26], [27], [28], [29], [30], [31], [32]]. The eligible articles were published between 2006 and 2023. Overall, 8 crossover trials [[18], [19], [20], [21], [22],[24], [25], [26],^28^,^31^] and 5 parallel trials [23,27,29,30,32] were included with a median duration of 28 d (range 10–180). A total of 553 participants were included in the reported analyses. Six trials were partial feeding studies with only red meat being provided to participants [[21], [22], [23], [24], [25], [26],^32^] and 7 trials were complete feeding studies [[18], [19], [20],^27^,^28^,^30^,^31^].

**TABLE 1:** Characteristics of the studies included.^1^

Study	Design and intervention delivery	Participants	Duration (d)	Intervention			Comparator		
				Red meat type	Red meat dose	Background diet	Protein type	Dose	Background diet
Cheung et al., 2017, United Kingdom [29]	Parallel, partial feeding (3 d/wk)	Healthy adults *n* = 50 Male: 50% Age: 59.4 ± 4.1 y BMI: 29.7 ± 2.6 kg/m^2^	21	Red meat	Week 1: 80 ± 13 g/d Week 2: 158 ± 35 g/d Week 3: 283 ± 46 g/d	—	Chicken: chicken breast	Week 1: 88 ± 10 g/d Week 2: 187 ± 16 g/d Week 3: 290 ± 75 g/d	—
							Processed meat: ham	Week 1: 98 ± 11 g/d Week 2: 259 ± 21 g/d Week 3: 483 ± 47 g/d	
							Fish: haddock	Week 1: 88 ± 10 g/d Week 2: 222 ± 36 g/d Week 3: 412 ± 42 g/d	
Crimarco et al., 2020, United States [21]	Crossover, partial feeding	Healthy adults *n* = 36 Male: 33% Age: 50.2 ± 13.8 y BMI: 27.9 ± 5.2 kg/m^2^	56	Beef, pork	2.6 ± 0.7 servings^2^/d	American	Nonanimal protein sources	2.5 ± 0.6 servings^2^/d	American
Djekic et al., 2020, Sweden [22]	Crossover, partial feeding	Adults with IHD *n* = 31 Male: 94% Age: 67 y (median) BMI: 28 ± 2.9 kg/m^2^	28	Beef, pork	145 g/d	Swedish	Nonmeat protein sources	—	Lacto-ovo-vegetarian
Dhakal et al., 2022, United States [20]	Crossover, complete feeding	Healthy adults > 50 y *n* = 36 Male: 28% Age: 66.4 ± 7.4 y BMI: 29.8 ± 5.6 kg/m^2^	10	Fresh lean pork	156 g/d	Dietary Guidelines for Americans	Chicken	156 g/d	Dietary Guidelines for Americans
Farsi et al., 2023, United Kingdom [31]	Crossover, partial feeding	Healthy adults *n* = 20 Male: 100% Age: 30.4 ± 7.92 y BMI: 24.0 ± 2.87 kg/m^2^	14	Red and processed meat (beef, pork)	240 g/d	United Kingdom	Mycoprotein Quorn products	240 g/d	United Kingdom
Krishnan et al., 2021, United States [19]	Crossover, complete feeding	Adults with overweight/obesity *n* = 39 Male: 31% Age: 46 ± 2 y (mean ± SEM) BMI: 30.5 ± 0.6 kg/m^2^ (mean ± SEM)	35	Beef, pork	71 g/d	Mediterranean	Red meat + poultry	29 g/d red meat	Mediterranean
Krishnan et al., 2022, United States [27]	Parallel, complete feeding	Adults with overweight/obesity *n* = 44 Male: 0% Age: 49.3 ± 11.2 y BMI: 31.6 ± 3.8 kg/m^2^	56	Beef, cold cuts, and sausage	88 ± 4 g/d	American	Beef, cold cuts, and sausage	26 ± 1 g/ d	Dietary Guidelines for Americans
Landry et al., 2023, United States [32]	Parallel, partial feeding	Healthy, identical twins *n* = 44 (22 twin-pairs) Male: 23% Age: 39.6 ± 12.7 y BMI: 25.9 ± 4.7 kg/m^2^	56	Beef, pork	(6–8 oz/d of meat, fish, or poultry)	Healthy omnivorous diet	Nonanimal protein sources	—	Healthy vegan diet
Porter Star et al., 2019, United States [23]	Parallel, partial feeding	Adults with obesity < 45y *n* = 80 Male: 10% Age: 64 ± 8 y BMI: 37.3 ± 6.6 kg/m^2^	180	Lean or very lean beef, pork	1.2 g/kg body weight (60g)	Hypocaloric	—	0.8 g/kg body weight	Hypocaloric
Schmedes et al., 2016, 2018, 2019, Norway [[24], [25], [26]]	Crossover, partial feeding	Healthy adults *n* = 20 Male: 35% Age: 50.6 ± 6.34 y BMI: 25.6 ± 0.7 kg/m^2^	28	Lean beef, pork	11.4% kcal from non-seafood	Norwegian nutrition recommendations	Lean seafood (cod, pollack, saithe, scallops)	11.4% kcal from seafood	Norwegian nutrition recommendations
Stella et al., 2006, United Kingdom [28]	Crossover, complete feeding	Healthy adults *n* = 12 Male: 100% Age: 25–75 y (range) BMI: –	15	Beef, pork	420 g/d	United Kingdom	Low meat diet: beef, pork	60 g/d	United Kingdom
							Vegetarian diet: nonmeat sources	420 g/d	
Tate et al., 2023, United States [30]	Parallel, complete feeding	Healthy adults ≥ 65 y *n =* 28 Male: 39% Age: 70.8 y (range: 65–84) BMI: 32 ± 6.9 kg/m^2^	84	Lean fresh beef	6 oz/d (170 g)	DASH diet	Lean fresh beef	3 oz/d (85g)	DASH diet
Wang et al., 2018, United States [18]	Crossover, complete feeding	Healthy adults *n* = 113 Male: 39% Age: 45 y (range: 21–65) BMI: 25.3 kg/m^2^ (range 18.2–35.3)	28	Beef, pork	12% kcal	American	White meat	12% kcal	American
							Nonmeat	16% kcal	

Abbreviation: IHD, ischemic heart disease.

1 Data are mean ± SD, unless otherwise stated.

2 Servings: burger (100 g), ground beef (100 g), “Good Morning” pork breakfast sausage (47 g), hot Italian sausage (71 g), pork bratwurst (57 g), and chicken breast (113 g).

The eligible publications included 15 comparisons of the effect of higher compared with lower red meat intake on TMAO concentrations. Specifically, in 5 comparisons, higher red meat intake was compared with a higher intake of plant-based protein sources [18,21,22,31,32]. In 4 comparisons, higher red meat intake was compared with higher poultry intake [[18], [19], [20],^29^]. One comparison examined higher red meat intake compared with higher nonmeat animal protein sources [23]. Three comparisons examined higher red meat intake compared with lower red meat-containing diets (replacement protein source not specified) [27,28,30], and 2 comparisons assessed higher red meat intake compared with higher fish intake [[24], [25], [26],^29^]. For 12 diet comparisons, TMAO concentrations were measured in serum or plasma [[18], [19], [20],^22^,^23^,^25^,^27^,^29^,^30^,^32^]. For 6 comparisons, urinary TMAO concentrations were reported [18,24,28,29,31], and for 1 comparison fecal TMAO concentration was reported [26]. For 1 diet comparison, plasma TMAO was measured, but between-diet differences in TMAO concentrations were not reported and therefore plasma results from this study are not presented [29]. Among the 15 comparisons, 10 used liquid chromatography-mass spectrometry (LC-MS)-based techniques to assess TMAO [[18], [19], [20], [21], [22],^27^,[29], [30], [31], [32]]. Nuclear magnetic resonance spectroscopy, a less sensitive but complementary method, was used to assess TMAO in 5 comparisons [[23], [24], [25], [26],^28^].

### Higher TMAO with higher red meat intake

In 6 comparisons, serum/plasma [18,19,21,30] and/or urine [18,28] TMAO concentrations were statistically significantly higher after higher red meat intake (∼71–420 g/d) compared with lower red meat intake in generally healthy adults after 15–84 d (Table 2) [[18], [19], [20], [21], [22], [23], [24], [25], [26], [27], [28], [29], [30], [31], [32]]. In 4 of these comparisons, urine and/or serum/plasma TMAO was higher after red meat intake (beef or pork) compared with plant-based protein [18,21] or poultry [18,19]. Two comparisons showed higher serum and/or urine TMAO concentrations after intake of a higher dose of red meat (mainly beef and pork) compared with a lower dose of red meat where the replacement protein source was not reported [28,30]. The leanness and processing level of the red meat included in many of these studies was not well-defined [18,28,29]. Wang et al. [18] reported higher estimated choline and carnitine intake during the higher red meat diet (choline: 573 mg/d; carnitine: 258 mg/d), compared with the lower red meat diets containing either plant-based protein sources (choline: 447 mg/d; carnitine: 22 mg/d) or white meat (choline: 498 mg/d; carnitine: 56 mg/d). Crimarco et al. [21] reported the mean difference in TMAO when comparing the higher red meat condition to the plant-based protein condition (2.0 μM, 95% CI: 0.3, 3.6); however, this effect should be cautiously interpreted because carryover effects were detected. Effect estimates were not reported for the other 5 diet comparisons.

**TABLE 2:**
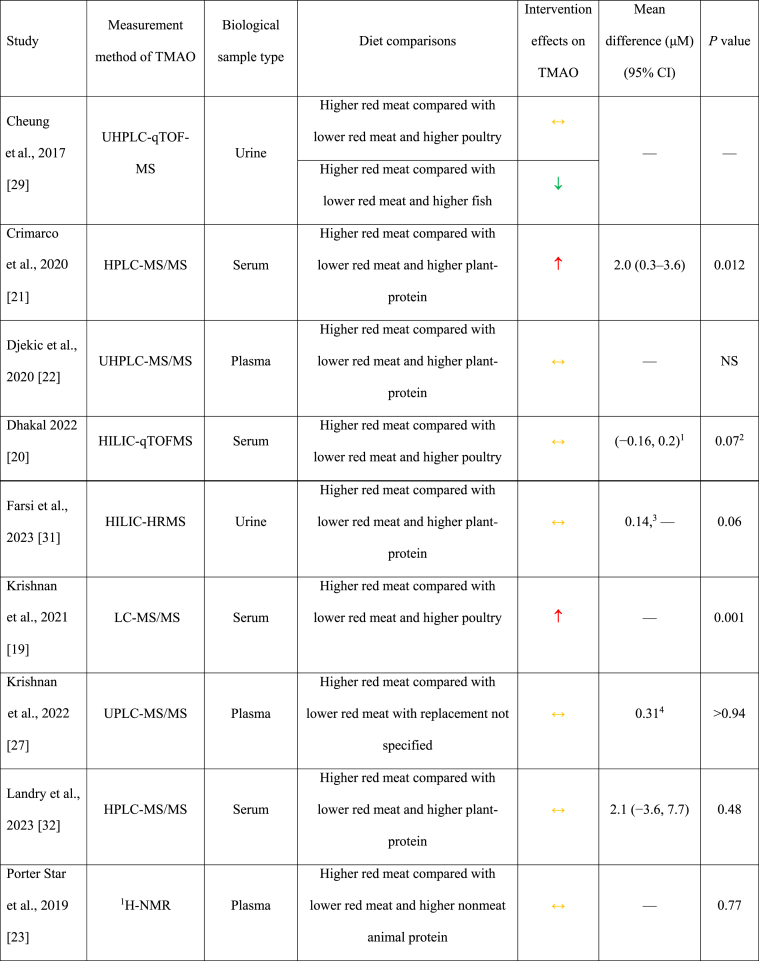

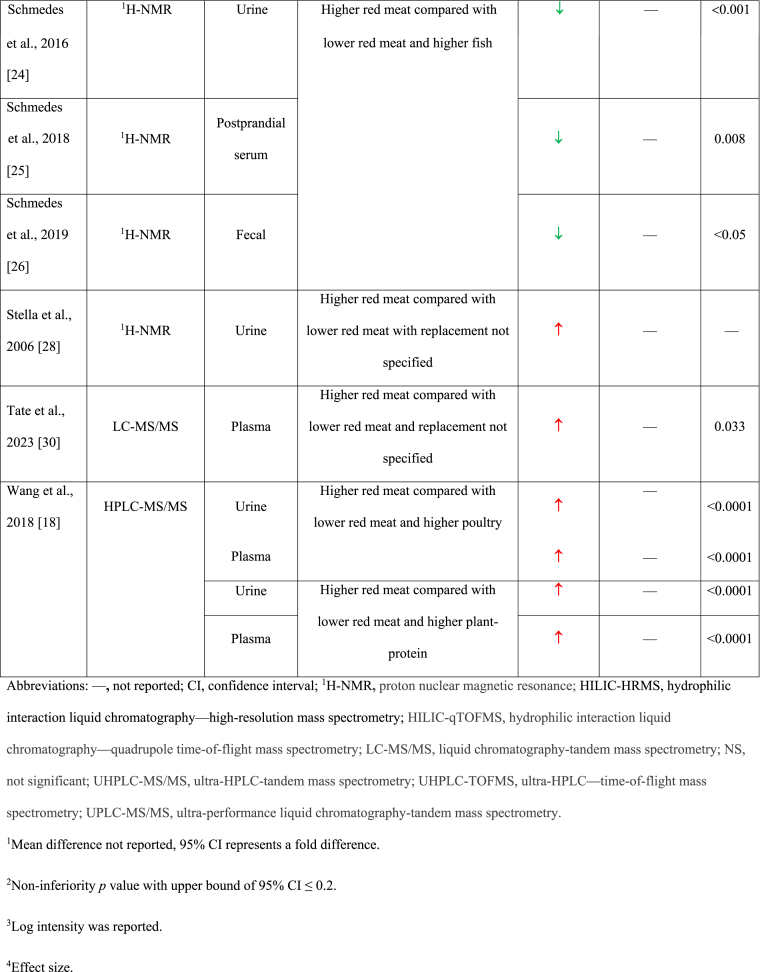
Summary of the results of the included studies.

### No effect of higher red meat intake on TMAO

In 7 diet comparisons, no significant differences in serum/plasma or urinary TMAO concentrations were observed after red meat consumption (beef or pork ∼60–240 g/d) compared with lower red meat diets containing nonmeat protein sources [22,23,31,32] or poultry [20,29], or lower red meat doses where the replacement protein source was not specified [27]. These studies were 10–180 d in duration and included females with overweight or obesity [23], individuals with ischemic heart disease [22], and generally healthy older adults with or without overweight and obesity [20,27,31,32]. In 2 studies, red meat was lean and/or very lean [20,23]; the remainder of the studies did not report the leanness and none of the studies reported the processing level.

For 2 of the comparisons, intake of dietary TMAO precursors was reported [20,27]. Krishnan et al. [27] reported that the dietary choline and carnitine content, assessed by chemical analysis of the 8-d menu, differed between the higher red meat diet (385.3 ± 10.4 mg/d and 40.3 ± 0.3 mg/d, respectively) and the lower red meat diet (459.0 ± 45.7 mg/d and 26.0 ± 0.6 mg/d, respectively). Dhakal et al. [20] assessed dietary intake of choline and carnitine from pork (138.8 mg/d and 40.3 mg/d, respectively) and chicken (123.2 mg/d and 5.5 mg/d, respectively) only using self-reported diet assessments.

### Lower TMAO with higher red meat intake

In 2 diet comparisons [[24], [25], [26],^29^], TMAO concentrations in urine, serum, and feces were lower after higher red meat intake (11.4% of total energy [[24], [25], [26]]; ∼ 174 g/d [29]) compared with diets lower in red meat and higher in lean seafood (11.4% of total energy [[24], [25], [26]]; ∼241 g/d [29]) in healthy adults after 21–28 d (Table 2) [[18], [19], [20], [21], [22], [23], [24], [25], [26], [27], [28], [29], [30], [31], [32]].

### Risk of Bias and GRADE assessment

Seven studies had a high risk of bias [18,21,[23], [24], [25], [26], [27], [28], [29]], 4 studies had some concerns of bias [19,20,22,30] and 2 studies had a low risk of bias (Table 3) [[18], [19], [20], [21], [22], [23], [24], [25], [26], [27], [28], [29], [30], [31], [32]]. Nine studies had some risk of bias concerns or high bias for the randomization process domain [[19], [20], [21], [22], [23], [24], [25], [26],[28], [29], [30]]. A high risk of bias from deviation from the intended intervention was present in 6 studies [18,[23], [24], [25], [26], [27], [28], [29]]. One study had a high risk bias from carryover effects because a wash-out period was not included [21]. All studies had a low risk of bias from the method used to measure TMAO. Only 1 study had concerns of bias from missing outcome data [28]. According to GRADE, the overall certainty of the evidence was rated as very low because of very serious concerns about risk of bias and serious concerns about inconsistency, indirectness, and imprecision (Supplemental Table 2).

**TABLE 3:**
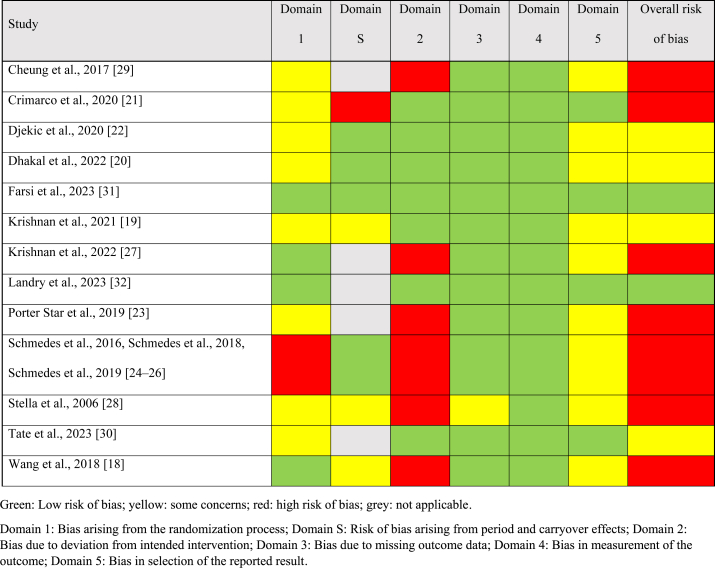
Summary of the risk of bias assessments for the included studies.

## Discussion

This systematic review was conducted to synthesize all the available RCT evidence on the effect of higher red meat intake, compared with lower red meat intake, on circulating, urinary, and fecal TMAO concentrations in generally healthy adults or adults with stable chronic diseases. In total, 15 publications reporting 13 RCTs and 15 diet comparisons were identified. Approximately half (*n* = 7) of the reported diet comparisons showed no difference in serum/plasma or urinary TMAO concentration with higher compared with lower red meat intake for a median of 28 d. In 6 comparisons, higher red meat intake increased circulating or urinary TMAO concentrations after a median of 35 d. In 2 diet comparisons, higher red meat intake decreased serum, urinary, and fecal TMAO concentrations compared with a lower red meat diet containing seafood. This systematic review demonstrates that there has been limited investigation of the effect of red meat on TMAO concentrations in RCTs and although some evidence suggests higher red meat intake increases circulating or urinary TMAO concentration, the magnitude of the effect and the clinical significance remain uncertain.

A recent umbrella review and meta-analysis of observational studies showed that higher TMAO concentrations are associated with an increased risk of CVD (relative risk (RR): 1.50; 95% CI: 1.26, 1.79), major adverse cardiovascular events (RR: 1.74; 95% CI: 1.56, 1.95), CVD mortality (RR: 2.02; 95% CI: 1.74, 2.34), and all-cause mortality (RR: 1.60; 95% CI: 1.43, 1.79) [33]. Although causation remains uncertain [6], evidence suggests TMAO increases ASCVD risk by promoting endothelial dysfunction, foam cell formation, thrombosis, and cholesterol metabolism impairment [34]. Our findings show that in 6 of the 15 reported diet comparisons, higher red meat intake, compared with lower red meat intake, increased TMAO concentrations. Effect estimates were only reported for one of these diet comparisons, which showed modestly higher TMAO concentration (2.0 μM, 95% CI: 0.3, 3.6) after higher red meat intake compared with intake of plant-based protein sources. However, caution is warranted because carryover effects were detected in this study [21]. Interestingly, 3 RCTs [19,21,30] reported absolute TMAO concentrations, and at baseline and following the dietary interventions, TMAO concentrations were <6.2 μM reflecting low concentrations that are not considered CVD risk enhancing [9]. Further research is needed to determine the clinical significance of red meat-induced changes in TMAO concentration.

The increase in TMAO observed with higher red meat intake in 6 diet comparisons [18,19,21,28,30] aligns with prior research showing that acute and chronic L-carnitine supplementation increases TMAO. Red meat is a dietary source of L-carnitine containing ∼24–122 mg/100 g [35,36]. In an RCT, oral supplementation with 1500 mg/d of L-carnitine for 24 wk in healthy elderly females led to a tenfold increase in fasting plasma TMAO concentrations [12]. Similarly, in an experimental study, intake of a 500 mg/d L-carnitine supplement increased fasting plasma TMAO after 2–3 mo [13]. Our findings of higher plasma/serum or urinary TMAO concentrations after higher intake of red meat, which contains L-carnitine, in 5 RCTs including 6 diet comparisons align with prior evidence on L-carnitine supplementation. However, in the majority of included studies, carnitine intake from red meat was not assessed; therefore, L-carnitine exposure in the included studies remains unclear.

In 7 of the 15 diet comparisons included in this systematic review, no difference in TMAO concentration was observed with a higher intake of red meat compared with a lower intake of red meat in the primary analyses [20,22,23,27,29,31,32]. For one of these comparisons, however, a sensitivity analysis where 3 outliers (2 at baseline and 1 at 8 wk) were removed, showed TMAO concentrations were higher (2.1 μM; 95% CI: 0.7, 3.5) with an omnivorous diet containing red meat compared with a vegan diet after 8 wk [32]. The 13 RCTs included in this systematic review are heterogenous in terms of the study methodology including the RCT design, intervention and comparator, TMAO assessment method as well as sample size. Thus, the reason(s) why red meat intake had mixed effects on TMAO across the included studies is not clear. Although previously it was shown in a cross-sectional analysis of individuals with metabolic syndrome-related risk factors/conditions that diet explained <5% of the variance in circulating TMAO concentrations; kidney function was the major determinant of circulating TMAO concentrations [37]. The studies included in our systematic review generally included healthy adults, and therefore it is likely that normal kidney function resulted in TMAO clearance, although kidney function was generally not reported, which may explain why red meat did not have a clear effect on TMAO.

It is also plausible that the inconsistent findings are related to the substantial day-to-day variability in TMAO, particularly in individuals with low TMAO concentrations (<6.2 μM). Wang et al. [18] found that the interday coefficient of variance was 0.43 for those with a TMAO concentration in the low range (median ∼3.5 μM), whereas when TMAO concentrations were >6.2 μM the interday coefficient of variance was 0.30. In 4 of the 7 studies where no difference in TMAO was observed with higher compared with lower red meat intake, reported TMAO concentrations at baseline and following the dietary intervention were low (<6.2 μM); the other 3 studies reported relative TMAO concentrations, which precludes comparison to the established reference range for TMAO. Therefore, it is possible that greater variance in TMAO resulted in a lack of power to detect statistically significant differences in generally healthy individuals. However, the clinical relevance of detecting small changes within the low range for TMAO needs to be considered because of the documented nonlinear relationship between TMAO and adverse outcomes [38]. Collectively, results from the short-term studies (median duration of 28 d), included in this systematic review suggest that red meat intake (∼71–420 g/d) may not increase TMAO concentrations to an extent that enhances CVD risk in generally healthy individuals with low baseline TMAO concentrations.

The clinical trials included in this systematic review examined the incorporation of red meat into a variety of different types of dietary patterns, which may also have contributed to the mixed TMAO findings observed. Previously, it has been demonstrated that TMAO concentrations are lower in vegans and vegetarians compared with omnivores [8,39]. Additionally, findings from animal research suggest that diet quality modifies the effect of red meat on TMAO generation. In a preclinical study, pigs had lower urinary TMAO excretion when red and processed meat was consumed as part of a prudent high-fiber, vegetable-rich background diet compared with when red and processed meat was consumed as part of a western background diet [40]. Three RCTs included in this systematic review examined red meat intake as part of a healthy diet. In 2 trials, higher red meat intake (71 and 170 g/d of beef and pork), compared with lower red meat intake (29 and 85 g/d), as part of a Mediterranean diet or a Dietary Approaches to Stop Hypertension diet increased TMAO concentrations [19, 30]. In an RCT examining the intake of a Dietary Guidelines for Americans adherent diet containing 156 g/d of pork compared with 156 g/d of chicken, no difference in TMAO was observed [20]. This divergent finding may be because pork contains less carnitine (∼24 mg/100 g) compared with beef (∼42–122 mg/100 g) [20,35,36]. Further examination of diet quality as a potential TMAO effect modifier is warranted because epidemiological evidence suggests diet quality modifies the association between TMAO and coronary heart disease risk. A prospective nested case-control analysis of the Nurses’ Health Study cohort demonstrated that high diet quality attenuated the association between TMAO and coronary artery disease risk, whereas low diet quality strengthened the association [41]. Diet quality may act through the gut microbiome to modify the relationship between TMAO and CVD. Gut microbiota play a crucial role in TMAO metabolism and are influenced by diet composition [[42], [43], [44]].

Gut microbiota composition was examined in 4 studies included in this systematic review [[20], [21], [22],^26^]. Three studies reported no difference in gut microbiota composition between high red meat diets and lower red meat diets with plant-based protein sources, poultry, and fish [[20], [21], [22]]. One study [26] showed microbiota composition differed by diet such that *Clostridium cluster IV* was decreased after the high red meat diet compared with the low red meat diet containing seafood. Schmedes et al. [26] also reported that following the higher red meat diet, circulating TMAO concentration was positively associated with 8 operational taxonomic units and inversely associated with 1 operational taxonomic unit. In contrast, Crimarco et al. [21] reported that no taxa predicted circulating TMAO concentrations. These findings align with evidence suggesting that microbiota composition does not consistently predict circulating TMAO concentrations because gene copy number does not predict bacterial metabolic activity [45]. This may be related to the regulation of transcription by substrate availability, translation regulation, posttranslation modifications, and cofactor availability. Further research is needed to understand regulators of gut microbial TMA production, including dietary influences, and influences on circulating TMAO concentrations.

The clinical trials included in this systematic review examined higher red meat-containing diets compared with a variety of different comparator diets containing various protein sources, which may also have contributed to the inconsistent findings. For lipids/lipoproteins, it is established that greater improvements occur when red meat is replaced with high-quality plant-protein sources (e.g., soy, nuts, and legumes) [46]. We did not observe any clear TMAO response patterns based on the comparator condition with the exception of fish. Two diet comparisons showed urinary, serum, and fecal TMAO was lower after a higher intake of red meat compared with diets lower in red meat and higher in fish [[24], [25], [26],^29^]. Fish and seafood contain free TMAO that is absorbed intact after fish consumption. Intake of fish rapidly and transiently increases circulating TMAO concentrations compared with foods containing TMAO precursors such as beef and eggs [47]. The effect of fish on TMAO conflicts with the well-established cardiovascular health benefits of fish intake [48]. It has been suggested that intake of seafood is not associated with increased CVD risk because of the significant variability in the TMAO content of fish as well as the transient nature of fish-related TMAO elevations [49]. This underscores the complexity of studying diet-related modulation of TMAO because endogenous TMAO production and exogenous TMAO exposure may be differentially associated with CVD risk.

A strength of this review is the inclusion of RCTs to examine the causal effects of red meat intake on TMAO concentrations. All of the included studies provided red meat and 7 studies were complete feeding trials where all of the food was provided therefore facilitating planned red meat exposure. This review is limited by the small number of RCTs that have examined the effects of intake of red meat on TMAO. Our review is also limited by the lack of effect size reporting in most of these studies, which precludes assessment of the clinical relevance of the findings. In addition, most studies included insufficient information about the intervention and comparator, including the red meat type, fat content, processing level, and composition of dietary TMA precursors. Furthermore, across the included studies, there was variability in the TMAO measurement methodology used, which may have contributed to result inconsistency. However, in 10 comparisons, LC-MS/MS was used, which is regarded as an established and validated analytical method for TMAO assessment because it has high sensitivity, specificity, and quantitative precision [50]. Finally, the included studies had a relatively short duration (median 28 d), which precludes assessment of long-term effects. However, previously it has been demonstrated that daily L-carnitine supplementation increased TMAO after 1 mo in omnivores with no further increase after 2 mo, suggesting TMAO stabilizes within 1 mo [18].

In this systematic review of 13 short-term RCTs including 15 comparisons, approximately half of the diet comparisons showed no difference in circulating or urinary TMAO concentrations with higher red meat intake compared with lower red meat intake. In 6 comparisons, higher red meat intake increased circulating or urinary TMAO concentrations. These mixed findings may be related to study methodology, interindividual variability in diet-related TMAO modulation, and/or the overall healthfulness of the red meat-containing diet. Further research investigating red meat-related TMAO modulation including the effect magnitude and clinical relevance, as well as contributors to interindividual variability is needed.

## Author contributions

The authors’ responsibilities were as follows – FJ, KSP: designed the research; KSP: primary responsibility for the final content; and all authors: involved in conducting the research, data interpretation, manuscript preparation, and read and approved the final manuscript.

## Data availability

Data described in the manuscript were extracted from the included published papers. These data are available for public access.

## Funding

This research was funded by the National Cattlemen’s Beef Association, a contractor to the Beef Checkoff. The funder has no role in the research design, project execution, data interpretation, or manuscript preparation. The funder has no publication restrictions.

## Conflict of interest

KSP received a grant from the National Cattlemen’s Beef Association to conduct the research. KSP has received honoraria from the National Cattlemen’s Beef Association for consulting work unrelated to the research presented in this article.
