# Supplementary material for: The Effect of Red Meat Consumption on Circulating, Urinary, and Fecal Trimethylamine-N-Oxide: A Systematic Review and Narrative Synthesis of Randomized Controlled Trials
Source: Adv Nutr. 2025 May 24;16(7):100453. doi: 10.1016/j.advnut.2025.100453 (PMC12273423; doi:10.1016/j.advnut.2025.100453)
Supplement: Multimedia component 1 [file mmc1.docx]

# ONLINE SUPPLEMENTAL MATERIAL

**Table of Contents**

**Supplemental Table 1**. Search Strategies for Each Database……………………………………..2

**Supplemental Table 2**. Certainty of evidence ratings using GRADE…………………………….3

**Supplemental References**………………………………………………………………………….3

## Supplemental Table 1. Search Strategies for Each Database

| **Database** | **Search Strategy** |
| --- | --- |
| PubMed | ("meat"[MeSH Terms] OR "meat"[All Fields] OR ("red meat"[MeSH Terms] OR ("red"[All Fields] AND "meat"[All Fields]) OR "red meat"[All Fields] OR "beef"[All Fields]) OR ("sheep"[MeSH Terms] OR "sheep"[All Fields] OR "lamb"[All Fields]) OR ("pork meat"[MeSH Terms] OR ("pork"[All Fields] AND "meat"[All Fields]) OR "pork meat"[All Fields] OR "pork"[All Fields]) OR ("goats"[MeSH Terms] OR "goats"[All Fields] OR "goat"[All Fields])) AND ("trimethyloxamine"[Supplementary Concept] OR "trimethyloxamine"[All Fields] OR "trimethylamine n oxide"[All Fields] OR ("trimethyloxamine"[Supplementary Concept] OR "trimethyloxamine"[All Fields] OR "tmao"[All Fields]) OR ("trimethyloxamine"[Supplementary Concept] OR "trimethyloxamine"[All Fields] OR "trimethylamine n oxide"[All Fields])) |
| Web of Science | (meat OR beef OR lamb OR pork OR goat)  AND  ("trimethylamine N-oxide" OR TMAO OR "trimethylamine-N-oxide") |
| Cochrane Collaboration Library | (meat OR beef OR lamb OR pork OR goat)  AND  ("trimethylamine N-oxide" OR TMAO OR "trimethylamine-N-oxide") |

## Supplemental Table 2. Certainty of evidence ratings using GRADE^[1]^

| **Study design; No. of articles** | **Risk of bias^1^** | **Inconsistency^2^** | **Indirectness^2^** | **Imprecision^2^** | **Publication bias^3^** | **Large effect** | **Plausible confounding** | **Dose-response** | **Summary of findings** | **Certainty** |
| --- | --- | --- | --- | --- | --- | --- | --- | --- | --- | --- |
| 13 RCTs from 15 publications | Very serious; High risk of bias in 9 RCTs and some bias concerns in 4 RCTs | Serious | Serious; 2 RCTs did not report red meat replacement in the comparator condition | Serious; 3 RCTs reported CIs, 2 reported mean difference | Undetected | N/A | N/A | N/A | Higher red meat intake for a median duration of 28 days had inconsistent effects on circulating and urinary TMAO concentration compared to lower red meat intake in generally healthy adults and/or adults with stable chronic disaease | Very low |

GRADE rating: Very low, low, moderate, or high.

^1^Domain only downgraded. Rating choices: extremely serious, very serious, serious, or not serious.

^2^Domain only downgraded. Rating choices: very serious, serious, or not serious.

^3^Domain only downgraded. Rating choices: strongly detected or undetected.

***Abbreviations***: CI, confidence interval; GRADE; Grading of Recommendations, Assessment, Development, and Evaluation; ROB, risk of bias; RCT, randomized controlled trial; N/A, not applicable

## Supplemental References

[1] Guyatt G, Oxman AD, Akl EA, Kunz R, Vist G, Brozek J, et al. GRADE guidelines: 1. Introduction-GRADE evidence profiles and summary of findings tables. *J Clin Epidemiol*. 2011;64(4):383-94. doi: 10.1016/j.jclinepi.2010.04.026.
